# Ultra Deep Sequencing of a Baculovirus Population Reveals Widespread Genomic Variations

**DOI:** 10.3390/v7072788

**Published:** 2015-07-07

**Authors:** Aurélien Chateigner, Annie Bézier, Carole Labrousse, Davy Jiolle, Valérie Barbe, Elisabeth A. Herniou

**Affiliations:** 1 Institut de Recherche sur la Biologie de l'Insecte, UMR 7261 CNRS-Université François Rabelais de Tours, Faculté des Sciences et Techniques, Avenue Monge-Parc Grandmont, 37200 Tours, France; E-Mails: aurelien.chateigner@univ-tours.fr (A.C.); annie.bezier@univ-tours.fr (A.B.); carole.labrousse@univ-tours.fr (C.L.); 2 Institut de Génomique, CEA, Génoscope, 2 rue Gaston Crémieux, 91057 Evry, France; E-Mail: vbarbe@genoscope.cns.fr

**Keywords:** genome population variation, quasispecies theory, AcMNPV, high-throughput sequencing

## Abstract

Viruses rely on widespread genetic variation and large population size for adaptation. Large DNA virus populations are thought to harbor little variation though natural populations may be polymorphic. To measure the genetic variation present in a dsDNA virus population, we deep sequenced a natural strain of the baculovirus Autographa californica multiple nucleopolyhedrovirus. With 124,221X average genome coverage of our 133,926 bp long consensus, we could detect low frequency mutations (0.025%). K-means clustering was used to classify the mutations in four categories according to their frequency in the population. We found 60 high frequency non-synonymous mutations under balancing selection distributed in all functional classes. These mutants could alter viral adaptation dynamics, either through competitive or synergistic processes. Lastly, we developed a technique for the delimitation of large deletions in next generation sequencing data. We found that large deletions occur along the entire viral genome, with hotspots located in homologous repeat regions (*hrs*). Present in 25.4% of the genomes, these deletion mutants presumably require functional complementation to complete their infection cycle. They might thus have a large impact on the fitness of the baculovirus population. Altogether, we found a wide breadth of genomic variation in the baculovirus population, suggesting it has high adaptive potential.

## 1. Introduction

Evolution relies on variation [1]. Most genetic mutations can be considered neutral or nearly neutral but few mutations are beneficial and confer a fitness advantage to the genome in which they occur and conversely genomes carrying deleterious mutations incur a fitness cost [2]. Natural selection should favor the genomes carrying beneficial mutations conferring the highest fitness, such that these best-adapted genomes remain at high frequency in a given population. When a new mutation occurs, there is little chance of it reaching high frequency in the population, as it can be eliminated through stochastic evolutionary events such as genetic drift [3].

Haploid and asexual diploid organisms are thought to incur a cost linked with clonality, which could hinder their adaptive response to changing environments [4,5]. However in large populations these mutational costs might be spread between individual genomes allowing the maintenance of lower fitness genotypes at low frequency, which can increase in frequency when suitable environmental conditions arise. It is also less likely that the fittest and most frequent genotype will be lost in large populations [6]. Large populations therefore have a higher probability of carrying pre-adapted genotypes allowing survival in changing environments [7].

Following primary infection by few infectious particles, virus populations increase rapidly but usually incur high mutation rates, which can bring deleterious mutations to individual genomes, but also beneficial mutations allowing the virus to evade from host immune response. Theory predicts virus populations, as a cloud of diverse mutational variants, should occupy larger sequence space in dynamic environments [8]. There are several lines of evidence showing that this occurs within the host [9]. However, there is little evidence that highly variable population structure might also be advantageous for transmission between hosts belonging or not to the same species [10]. There are ample examples of highly diverse viral population for small RNA or DNA viruses [11,12], but there is little evidence that it could be generalized to all viruses, including large double stranded DNA (dsDNA) viruses. Deep sequencing of varicella-zoster virus showed that this large dsDNA virus does evolve within human hosts [13,14]; however, the amount of variation found in the population remained relatively small, probably because of the relative homogeneity of the inoculum and of the analyzed sample size under immune suppression. To explore the potential breadth of viral diversity, one would need to study the genetic variability within a large DNA virus population. Recent studies found that the human cytomegalovirus intrahost population was as genetically diverse as RNA viruses [15,16]. However, to what extent, and with what impact on fitness, could this genetic diversity be preserved and transmitted after systemic infections?

**Figure 1:**
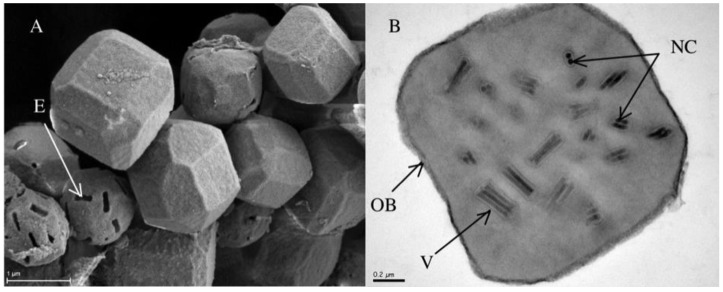
*Autographa californica multiple nucleopolyhedrovirus* (AcMNPV) occlusion bodies. (**A**) Scanning electron microscopy (×20,000) showing several occlusion body (OB) shapes. The silhouette of virions is visible on emptied OBs (E); (**B**) Transmission electron microscopy (×50,000) showing the cross section of one OB with rod shape virions (V) and nucleocapsids (NC).

Baculoviruses are large dsDNA viruses disseminated in the form of occlusion bodies (OBs, Figure 1) harboring dozens of virions [17], each of which can enclose multiple nucleocapsids [18], themselves containing one circular viral genome. Baculoviruses infect insect hosts through the ingestion of contaminated food plants. Infections are typically initiated by OBs and thus by populations of genomes. Though assembled within infected cells, these genomes found in single OB are not necessarily clonal, due to possible mixed infections of the cells [19], intra particle variation [20] and to mutations occurring during replication [21]. A single *Panolis flammea* caterpillar collected in the wild was indeed found to contain 24 baculovirus genotypic variants based solely on restriction fragment length polymorphism [22]. Deleterious genotypes may also be maintained over several infection cycles by complementation within OBs containing wild type genomes [23] and may even increase viral population fitness [24].

*Autographa californica multiple nucleopolyhedrovirus* (AcMNPV) is the best-characterized baculovirus [25] that has been defined as the type species of the genus *Alphabaculovirus* [26]. It displays the typical morphology of baculoviruses, *i.e*., OBs harboring numerous virions themselves containing around three rod-shaped nucleocapsids (Figure 1). AcMNPV has a circular dsDNA genome of ~134 kb [25] encoding 151 ORFs [27], including 37 core genes shared by the entire *Baculoviridae* family [28]. AcMNPV has a broad host range spanning several lepidopteran families [29,30] and thus has to be able to adapt to changing environments (*i.e*., hosts). Seven genomic variants have been identified by restriction analyses [20,31,32,33], but so far the true extent of the genetic diversity present within baculovirus populations remains unknown. Here we determined the genetic variation present in a natural population of AcMNPV, which we named AcMNPV-WP10 (WP10) and was reported to contain one moth transposable element in ~8500 AcMNPV genomes [34]. We used ultra-deep sequencing to gain insights on the functional diversity encoded by this virus, its genetic structure, and on its adaptive potential.

## 2. Materials and Methods

### 2.1. Virus Amplification and DNA Extraction

The baculovirus AcMNPV was first isolated in 1964 from a single field caterpillar of the alfalfa looper (*Autographa californica*) by Crumb’s methods [35,36]. The AcMNPV-WP10 isolate (Wild Population 2010) used in the present study was obtained by *in vivo* amplification of an archival sample of the original AcMNPV isolate through a one-cycle infection of 500 highly susceptible cabbage looper (*Trichoplusia ni*) caterpillars using the diet plug method [37]. Individual caterpillars were fed approximately 4000 OBs per 5 mm^3^ diet plug. Viral amplification conditions, *i.e*., highly susceptible hosts caterpillar species and high viral dose [30], were chosen so as to minimize selection on viral genomes. Upon host death, OBs were first filtered through cheesecloth, purified twice by centrifugation (10 min at 7000 rpm) with SDS 0.1% then distilled water, and finally resuspended in water. Approximately 1.5 × 10^10^ OBs were treated as described in [34] to provide ~80 µg of high quality viral dsDNA (~5.82 × 10^11^ genomes).

### 2.2. Sequencing, Consensus Genome Assembly and Annotation

A paired-end library was constructed by sonicating 2 µg of purified viral dsDNA (1.47 × 10^10^ genomes of ~134 kb) to a 100-to-800 bp size range using the E210 Covaris instrument (Covaris, Woburn, MA, USA). Fragments were end-repaired, then 3’-adenylated, and Illumina adapters were added by using NEBNext Sample Reagent Set (New England Biolabs, Ipswich, MA, USA). Ligation products were purified by Ampure XP (Beckman Coulter, Fullerton, CA, USA) and DNA fragments (>200 bp) were PCR-amplified using Illumina adapter-specific primers and Platinum^®^ Pfx DNA polymerase (Invitrogen, Carlsbad, CA, USA). Amplified library fragments were size selected on 1.5% agarose gel at 260 bp. After library profile analysis by Agilent 2100 Bioanalyzer (Agilent Technologies, Santa Clara, CA, USA) and qPCR quantification (MxPro, Agilent Technologies), each library was sequenced using 151 bp-length read chemistry in a paired-end flow cell on the HiSeq™ 2000 sequencing system (Illumina, San Diego, CA, USA).

Two approaches were conducted to assemble the produced 1.71 × 10^8^ paired-end reads (Genbank accession number SRS533250). On the one hand, *de novo* assembly using the Newbler 2.8 program [38] was carried out with the following parameters: 90% minimal overlap identity and 20 to 25 bases of minimal overlap length. On the other hand, all the reads were mapped on the AcMNPV-C6 (C6) genome (accession number NC_001623) using the bwa software [39]. The second approach allowed bridging of the nine homologous repeat regions (*hrs*), which could not be resolved with the *de novo* assembly strategy. We manually compared with Geneious 8 the large contigs from the *de novo* assembly to the sequence from the mapping, to order the contigs, complete the misassembled sequence and finally generate the WP10 consensus genome (accession number KM609482). ORFs were predicted with Geneious and corrected by comparison with the C6 annotation and BLAST [40]. The WP10 annotation was translated from an xml formatted blast output file into Genbank format using the Blast2Gb.pl software [41].

### 2.3. Mutation Detection and Analyses

To detect polymorphism within the WP10 genome population, 1.50 × 10^8^ paired-end reads, with at least 100 consecutive bases of Phred quality score above 30 (99.9% base call accuracy, bases below this threshold were trimmed), were re-mapped on the AcMNPV-WP10 consensus sequence using the bowtie 2 software [42]. Single nucleotide polymorphisms (SNPs) and short insertions and deletions (indels) were detected by using SAMtools mpileup [43]. We previously showed that a genome represented by a single read could be PCR amplified and Sanger sequenced [34]. However, to take into account possible experimental errors linked to Illumina sequencing, we set a mutation frequency above the error rate (>10^−3^ for all the possible changes, or >2.5 × 10^−4^ per nucleotide) as threshold to the genuine detection of variation. Geneious (versions 6.1.7 and 8.0.5) was used to visually validate variations. The repartitions of the SNPs in different frequency groups was assessed by k-means clustering [44] with the R function “kmeans” [45]. K-means computations, whereby each SNP was addressed to a cluster, were iterated 100 times with and without shuffling of the reads between iterations. The final assignment of a given SNP to a particular cluster corresponds to the consensus of the individual k-means classifications. To represent the k-means clusters, violin plots were drawn with the “vioplot” R package [46], combining the basic summary statistics inherent to box plots with the information available from local density estimates. To discriminate between positions evolving under non-random *versus* neutral processes, Tajima’s D statistics [47] have been calculated at each position of the genome using a homemade script (available upon request).

### 2.4. Detection of Large Deletions

**Figure 2:**
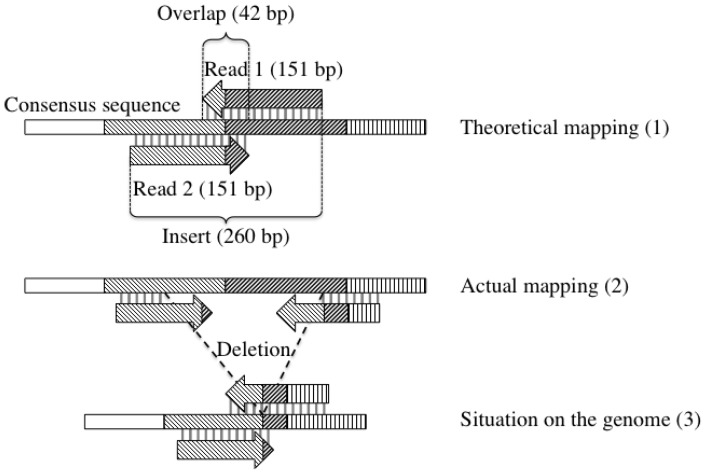
Strategy for finding large deletions. A genome consensus sequence section is shown along with the two pair reads associated with it and the position they map depending on the analyses conducted. Grey bars between reads and the consensus sequence represent a good alignment. (**1**) Read 1 and Read 2 represent paired-end reads, they should theoretically map on the consensus sequence with an overlap of about 42 bp as the insert is 260 bp long; (**2**) The actual mapping with a larger distance between reads of a pair and/or a poor mapping of the end of the reads can differ from expectation based on the consensus; (**3**) This different mapping happens because the genome from which the reads were produced carried a deletion.

We developed a new approach to detect the boundaries of large deletions based on the analysis of the distance between pairs of Illumina paired-end reads. The theoretical size of the Illumina insert library is 260 nucleotides; the 151 bp paired-end reads are therefore expected to overlap by about 42 nucleotides (Figure 2). When mapping the paired-reads on the consensus, the reads that do not have the expected overlap are rejected even if each read individually can map to the consensus sequence. We developed a script, called largeDeletionsExtractor.sh, to remap individually all the reads, by omitting pair information, using a Phred quality score above 30 (5,389,378 reads) to avoid any mapping error due to poor read quality. The pair information was then reinstated and linked to the mapped position of the reads so as to calculate the length of the gap separating them on the consensus sequence. The distribution of gap length was then plotted on the sequence to determine which read pairs were the most distant to the mean. To avoid any potential sequencing method artifact and since the distance distribution is close to a normal distribution, only the 5% and 2.81% most distant pair of reads were selected and extracted to study their location on the genome. These reads give us the boundaries of the 5% and 2.81% largest genomic deletions found in the viral genome population.

The presence of a large deletion between *hr5* and *hr1* in the population has been verified by Sanger sequencing. First a 25 µL amplification reaction was performed from 1 ng purified WP10 DNA by using 0.5 pmol µL^−1^ of each primer (hr5hr1-F: CTACAGAATCGAGCTGGGGC; hr5hr1-R: TCTTCGCTAGTCACGTACGC), 3 mM MgCl_2_, 0.2 mM dNTP and 0.75 unit Diamond Taq polymerase (Eurogentec) under a 30-cycles PCR program (95 °C for 4 min; 30 cycles of 95 °C for 60 s, 60 °C for 60 s, 72 °C for 60 s, and 72 °C for 10 min). Then the PCR product was purified using the NucleoSpin^®^ Gel and PCR Clean-up kit (Macherey-Nagel, Düren, Germany) and sequenced on ABI PRISM 3100-Avant system using the BigDye Terminator kit according to manufacturer’s instructions (Life Technologies, Grand Island, NY, USA).

## 3. Results and Discussion

### 3.1. AcMNPV-WP10 Genome Sequence and Annotation

We sequenced a genome population of the baculovirus AcMNPV-WP10 (WP10). This WP10 isolate was obtained by amplifying an archival sample of the original AcMNPV Vail isolate [36] through a one-cycle *in vivo* infection under permissive conditions. The sequencing effort amounted to 1.71 × 10^8^ Illumina paired-end reads. Following *de novo* assembly, we obtained a WP10 consensus sequence of 133,926 bp with an overall A + T content of 59.3%. In total, 1.50 × 10^8^ paired-end reads could be mapped on this genome, resulting in an extremely deep and uniform mean coverage of 124,221X (sd = 19,391X). As around 1.47 × 10^10^ genomes of ~134 kb were nebulized (2 µg DNA, AcMNPV genomic DNA weights = 1.36 × 10^−4^ pg [48]), and randomly sequenced, each pair of reads probably derives from a different molecule (5.1 × 10^−3^ odds that two pairs come from the same genome).

**Table 1:** Sequence variations observed between AcMNPV-WP10 consensus sequence and AcMNPV-C6 genome.

WP10/C6 sequence ^a^	Position on WP10 ^b^	Position on C6 ^c^	Type	WP10 gene ^d^	C6 gene
-/A	9707	14,226	Indel	*Ac17*	*Ac17*
G/A	11,651	16,171	SNP	*Ac20/21*	*Ac20*
CG/-	11,685	16,205	Indel	*Ac20/21*	*Ac20*
AC/-	11,690	16,208	Indel	*Ac20/21*	*Ac20*
G/C	11,692	16,208	SNP	*Ac20/21*	*Ac20*
-/G	11,698	16,214	Indel	*Ac20/21*	*Ac20*
-/C	11,788	16,305	Indel	*Ac20/21*	*Ac21*
-/G	38,855	44,372	Indel	*Ac52*	*Ac52*
G/T	43,197	47,714	SNP	*Ac58/59*	*Ac58*
A/-	43,398	47,914	Indel	*Ac58/59*	*Ac59*
-/CGACGGTCGAGGG	67,379	71,893	Indel	Non-coding ^e^	Non-coding ^e^
-/TATAATTTTT	69,604	74,134	Indel	Non-coding^e^	*Ac86*
A/-	89,218	93,749	Indel	*Ac106/107*	*Ac106*
A/-	89,288	93,818	Indel	*Ac106/107*	*Ac106*
C/-	89,326	93,865	Indel	*Ac106/107*	*Ac106*
CA/-	89,414	93,953	Indel	*Ac106/107*	*Ac106*
C/A	89,417	93,954	SNP	*Ac106/107*	*Ac106*
G/-	89,447	93,983	Indel	*Ac106/107*	*Ac106*
CG/-	89,497	94,033	Indel	*Ac106/107*	*Ac106*
A/G	89,573	94,107	SNP	*Ac106/107*	*Ac107*
ATTTGG/-	89,576	94,110	Indel	*Ac106/107*	*Ac107*
A/-	89,587	94,114	Indel	*Ac106/107*	*Ac107*
-/A	92,249	96,777	Indel	*Ac112/113*	*Ac112*
G/A	92,440	96,968	SNP	*Ac112/113*	*Ac113*
T/C	92,635	97,163	SNP	*Ac112/113*	*Ac113*
C/T	92,885	97,413	SNP	*Ac112/113*	*Ac113*
T/C	92,998	97,526	SNP	*Ac112/113*	*Ac113*
G/A	93,065	97,593	SNP	*Ac112/113*	*Ac113*
G/-	107,127	111,645	Indel	*Ac131*	*Ac131*
-/T	120,584	125,113	Indel	*Ac143*	*Ac143*
-/T	120,586	125,116	Indel	*Ac143*	*Ac143*
-/A	121,748	126,790	Indel	*Ac145*	*Ac145*
ATCTG/-	133,286	3919	Indel	*Ac7*	*Ac7*
TATTT/-	133,602	4229	Indel	*Ac7*	*Ac7*
AACAACGCTGCAT/-	133,610	4232	Indel	*Ac7*	*Ac7*
ACATTA/-	133,625	4234	Indel	*Ac7*	*Ac7*
ATTTCGGCTT/-	133,808	4411	Indel	Non-coding ^e^	Non-coding ^e^

^a^ Nucleotide variation between the WP10 consensus sequence, on the left of the slash, and the C6 sequence, on the right; ^b^ Position on the WP10 consensus sequence; ^c^ Position on the C6 sequence. The WP10 and the C6 sequences are not starting at the same locus, the WP10 starts at the ATG of the *polh* gene (4520th base of the C6 sequence), the C6 starts at the *hr1* (and ends at the 129,373th base of WP10 consensus sequence); ^d^ Gene found on the WP10 consensus sequence, can differ from the C6 sequence when the variation changes the sequence and removes a stop codon, stretching out the open reading frame to the next gene end, or when a stop codon is inserted, stretching in the open reading frame to the next ATG codon; ^e^ Non-coding means that the variation is located in a non-coding sequence, it can happen after a change in the open reading frame.

**Figure 3:**
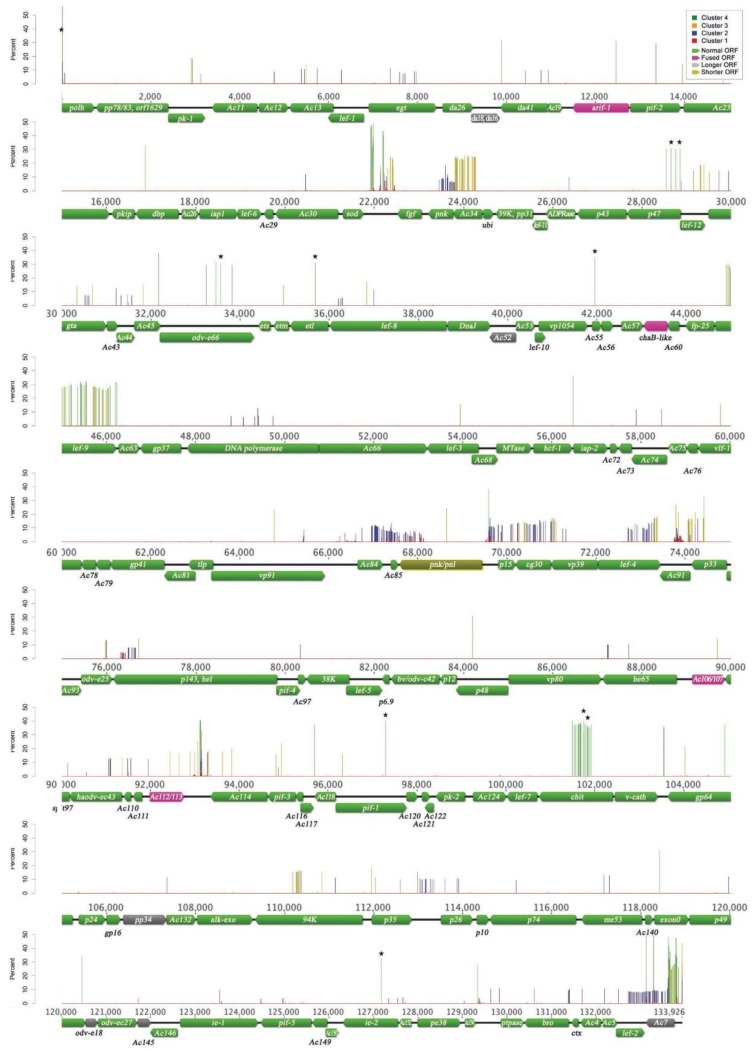
Location and frequency of non-consensus single nucleotide polymorphisms (SNPs) on the AcMNPV-WP10 consensus genome. The WP10 consensus genome is presented as a linear map. Arrows indicate the transcriptional direction of predicted ORFs. Arrows are colored according to the comparison of the WP10 and C6 AcMNPV genomes (green: ORFs with identical size in both genomes; pink: ORF fusion; grey: longer ORF, yellow: shorter ORF; see Table 1 for details). Non-consensus SNPs are plotted as frequency at the locus they were identified with a color corresponding to the k-means cluster they belong to (Table 2). Stars highlight cluster 4 SNPs changing amino acid polarity.

We annotated 151 ORFs (Figure 3). Our consensus genome is 99.8% similar to that of the AcMNPV-C6 clone (C6) [25], also deriving from the Vail isolate. The main differences in terms of ORFs concern the fusions of adjacent C6 ORFs (*Ac20/Ac21*, *Ac58/Ac59*, *Ac106/Ac107*, *Ac112/Ac113*) (Figure 3 and Table 1), as previously reported [27]. However, only the fusion between ORFs 106 and 107 (*Ac106/107*) is supported by a recent transcriptomics study [49], showing both ORFs share the same transcription starting site (TSS). For all the other fusions different TSS positions for each ORFs were determined at different infection time points [49]. This suggests all the other fused ORFs (*Ac20/21* = *arif-1*, *Ac58/59* = *ChaB-like*, *Ac112/113*) might be alternatively spliced ORFs. WP10 consensus genome analysis also reveals some ORFs longer than reported for the C6 clone: *Ac17 (da18)*, *Ac52*, *Ac131* (*pp34*), *Ac143* (*odv-e18*) and *Ac145.* However, *Ac86* (*pnk/pnl*) is 221 bp shorter. These variations are due to single nucleotide polymorphisms (SNPs) and short indels (insertion/deletion), changing the positions of stop codons for *Ac17*, *Ac52* and *Ac131* and the position of the first methionine for *Ac143* and *Ac145*. These observed variations are all compatible with the TSS found by transcriptomics [49].

### 3.2. Nucleotide Variation in the AcMNPV-WP10 Genome Population

To study in depth the genetic variation present within the WP10 genome population, we mapped 1.50 × 10^8^ paired-end reads with quality scores >30 on the WP10 consensus sequence. The mean sequence coverage for these analyses was 124,221X allowing at each position the significant detection of mutations present at a frequency higher than 2.5 × 10^−4^, above the sequencing error rate for any nucleotide. The accuracy and quality of the dataset was previously attested by PCR validation of the extremely rare insertion of transposable elements detected in single reads [34]. We detected 3243 SNPs with a frequency higher than 2.5 × 10^−4^ on our 133,926 bp long sequence, found in reads positioned in both orientations of genome and supported by both reads of a pair. When looking at the variation below 2.5 × 10^−4^ in frequency, we found all the possible mutations at each position, but as these SNP calls could be confused with sequencing error they were discarded. This suggests AcMNPV presents a high adaptive potential, as mutations that could bring a large fitness benefit in a different host are potentially already present in the population. The frequency of such mutation would determine how readily the viral genome population is functionally pre-adapted.

**Figure 4:**
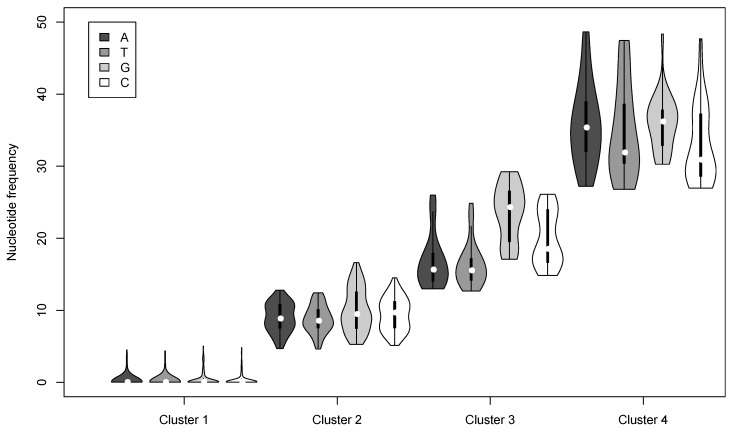
Frequency of K-means clusters. For each violin plot, the white dot represents the median, the black bar limits represent the 1st and 3rd quartile (respectively the lower and the upper limit of the bar). The shapes of the violins represent the probability density of the nucleotides in the cluster for the different percentages. The color of the shading represents the nucleotide as shown in the legend (always in the order A: Adenine, T: Thymine, G: Guanine, C: Cytosine).

To assess whether there were different groups of mutational variants (genotypes) that can be identified from the consensus sequence of our viral genome population, we performed a k-means clustering analysis on the SNPs. We obtained four clusters corresponding to groups of SNPs with similar frequencies (Figure 3 and Figure 4, Table 2). These results are robust as they were obtained from 100 computations, and as at least 98% of the iterations provided the same clustering, whether or not the reads were shuffled. Cluster 1 grouped together the vast majority (over 78%) of SNPs but with a mean frequency of only 2.7 × 10^−3^ mutations per nucleotide in the population. These SNPs are in extremely low frequency but are genuine as they are covered by more than 0.025% reads with 99.9% base call accuracy. Cluster 2 grouped over 12% of mutations with a mean frequency of 9.3 × 10^−2^. Cluster 3 corresponds to 5% of the mutations with an average frequency of 0.19. Cluster 4 grouped together only 118 SNPs (3.6% of the variations) but with the highest frequency of 0.35 within our AcMNPV genome population (Table 2). These different groups of mutations seem to belong to different biological classes present in one species, which could impact differently the evolution of the viral population. SNPs could be attributed to the same genome only when found within a 260 bp distance (length of our sequencing insert). Other variations further apart might possibly be linked on the same genome if found at the same frequency in the population. However, in the absence of long sequencing reads experimentally linking these SNPs, we chose to avoid speculation.

Considering an infectious baculovirus particle (*i.e*., OB) contains over ten virions, each enclosing around three nucleocapsids (*i.e*., genomes), thus each OB contains around 30 genomes 133,926 bp long (4,017,780 bases), by multiplying each SNP frequency by the number of bases, we found that each genome carries around 94 SNPs of any clusters and that each OB carries around 2815 mutations. However, even if cluster 1 mutations are in a relatively high number in the population when compared to the other clusters, their frequency at each site is the lowest (2.7 × 10^−3^). There is therefore little chance for each SNP from cluster 1 to be carried forward to the next generation. Thus, they likely reappear from mutations at each generation. Therefore, we propose that their mean frequency, 2.7 × 10^−3^ mutations *per* nucleotide, *per* infection cycle, might represent the mutation frequency of the population, that is closer to the RNA viruses mutation rate (1.5 × 10^−3^ mutations *per* nucleotide, *per* genomic replication) than to the DNA viruses’ (1.8 × 10^−8^ mut/nt/rep) [50]. We have, however, estimated a mutation frequency per *in vivo* infection cycle and not per genomic replication and it is thus difficult to compare to previous mutation rates studies due to differences in scale and variation between hosts. We cannot apply the fluctuation test of Luria and Delbruck [51] because it implies clonally expanding populations, and natural AcMNPV populations are hardly clonal. The other method commonly used for estimating mutation rate is the mutant accumulation [52], which is not applicable in this case. Last, the sequencing quality of the reads, although above Q30, may artificially increase the mutation frequency and higher quality sequencing might in the future lower this estimation.

The variations found in clusters 4 and 3 have the second and third highest frequencies in the genome population (0.35 and 0.19). From these 282 SNPs, 73 are found in non-coding regions. Although none were found in the TSS regions [49], these mutations could be adaptive for example by enhancing or down-regulating gene expression. Interestingly, we found six of these mutations close to the TSS of *iap-2* (distance = 15 nt), *p15* (1 nt), *Ac91* (16 nt), *Ac109* (14 nt), *p94* (11 nt) and *Ac7* (3 nt). Of 209 SNPs found in coding regions, only 105 are non-synonymous, 60 of which involve a change in the amino-acid polarity [53]. They are classified in several functional gene classes: accessory, host interaction, budded virus (BV) specific, occlusion-derived virus (ODV) specific, packaging and assembly (associated to both BV and ODV), replication and transcription, as well as in genes of unknown function. Forty of these mutations were linked two by two on the same reads and thus on the same genome (Table 3).

**Table 2:** Statistics of the non-consensus SNP clusters.

Cluster	Nucleotide	Number of loci	Mean Frequency ^a^	# *per* genome ^b^	# *per* OB ^c^
1	A	903	0.23	1.39	137.71
	T	969	0.20	1.44	
	G	328	0.44	0.87	
	C	361	0.36	0.89	
2	A	98	8.97	7.07	933.75
	T	104	8.77	7.41	
	G	112	10.02	9.79	
	C	86	9.43	6.86	
3	A	39	16.75	5.94	842.56
	T	41	16.31	6.35	
	G	46	23.59	9.72	
	C	38	19.92	6.07	
4	A	25	35.82	6.03	901.33
	T	25	34.86	5.10	
	G	30	35.99	7.95	
	C	38	32.91	10.96	

^a^ Mean frequency for all loci (in percent); ^b^ Number *per* genome; ^c^ Number *per* OB.

**Table 3:** Characteristics of the SNPs found in the clusters 3 and 4 on AcMNPV-WP10 genome population leading to a change in amino-acid polarity.

Nt ^a^	Gene function ^b^	Position ^c^	Perc. ^d^	Cluster	Gene ^e^	Codon Change	AA change	AA class change ^f^
C	Accessory	101,605	37.55	4	Ac126/chitinase	TCA->GCA	S->A	NP->No polarity
C	Accessory	101,638	38.05	4	Ac126/chitinase	AAA->GAA	K->E	BP->AP
T	Accessory	101,647	38.08	4	Ac126/chitinase	CCC->ACC	P->T	No polarity->NP
G	Accessory	101,716	40.18	4	Ac126/chitinase	GGT->CGT	G->R	No polarity->BP
G	Accessory	101,793	36.25	4	Ac126/chitinase	AGA->AGC	R->S	BP->NP
A	Accessory	101,851	35.62	4	Ac126/chitinase	GGC->TGC	G->C	No polarity->NP
C	Accessory	101,884	37.25	4	Ac126/chitinase	TCA->GCA	S->A	NP->No polarity
T	Host interaction	110,347	15.71	3	Ac134/p94	CGC->AGC	R->S	BP->NP
G	BV specific	14,832	19.28	3	Ac23/f-protein	ACA->GCA	T->A	NP->No polarity
T	BV specific	14,833	18.27	3	Ac23/f-protein	ACA->ATA	T->I	NP->No polarity
A	BV specific	103,990	21.18	3	Ac128/gp64	TCG->TTG	S->L	NP->No polarity
A	ODV specific	2	36.71	4	Ac8/polyhedrin	ATG->AAG	M->K	No polarity->BP
A	ODV specific	4	38.94	4	Ac8/polyhedrin	CCG->ACG	P->T	No polarity->NP
T	ODV specific	4	32.48	4	Ac8/polyhedrin	CCG->TCG	P->S	No polarity->NP
A	ODV specific	5	12.99	3	Ac8/polyhedrin	CCG->CAG	P->Q	No polarity->NP
A	ODV specific	13,917	14.02	3	Ac22/pif-2	GGG->AGG	G->R	No polarity->BP
A	ODV specific	33,571	30.25	4	Ac46/odv-e66	GCC->ACC	A->T	No polarity->NP
G	ODV specific	64,772	22.98	3	Ac83/vp91	ACC->GCC	T->A	NP->No polarity
A	ODV specific	70,144	13.09	3	Ac88/cg30	CAA->AAA	Q->K	NP->BP
A	ODV specific	70,234	13.58	3	Ac88/cg30	GCC->TCC	A->S	No polarity->NP
A	ODV specific	70,268	13.58	3	Ac88/cg30	ACA->ATA	T->I	NP->No polarity
A	ODV specific	70,381	13.68	3	Ac88/cg30	GAC->TAC	D->Y	AP->NP
T	ODV specific	70,399	13.78	3	Ac88/cg30	GCG->ACG	A->T	No polarity->NP
G	ODV specific	94,932	23.84	3	Ac115/pif-3	GGT->CGT	G->R	No polarity->BP
C	ODV specific	97,271	40.46	4	Ac119/pif-1	TCG->CCG	S->P	NP->No polarity
T	PA	71,103	14.86	3	Ac89/vp39	CGC->AGC	R->S	BP->NP
C	PA	74,220	21.51	3	Ac92/p33	AAA->GAA	K->E	BP->AP
C	PA	74,391	17.50	3	Ac92/p33	AAA->GAA	K->E	BP->AP
A	PA	74,421	32.40	4	Ac92/p33	CAC->TAC	H->Y	BP->NP
G	PA	84,226	30.75	4	Ac103/p45	ACT->CCT	T->P	NP->No polarity
T	PA	91,064	13.25	3	Ac109/odv-ec43	GAG->AAG	E->K	AP->BP
A	Replication	29,534	13.78	3	Ac42/gta	GCG->ACG	A->T	No polarity->NP
A	Transcription	28,878	30.16	4	Ac41/lef-12	GAC->AAC	D->N	AP->NP
G	Transcription	29,337	18.47	3	Ac41/lef-12	ACA->GCA	T->A	NP->No polarity
T	Transcription	29,346	18.55	3	Ac41/lef-12	CCA->TCA	P->S	No polarity->NP
A	Transcription	36,848	17.11	3	Ac50/lef-8	CAC->TAC	H->Y	BP->NP
G	Transcription	45,913	26.33	3	Ac62/lef-9	AAA->GAA	K->E	BP->AP
C	Transcription	68,643	24.53	3	Ac86/pnk/pnl	ACA->GCA	T->A	NP->No polarity
T	Transcription	73,303	16.73	3	Ac90/lef-4	TCG->TTG	S->L	NP->No polarity
T	Transcription	73,356	16.95	3	Ac90/lef-4	CCG->TCG	P->S	No polarity->NP
G	Unknown	23,830	24.44	3	Ac34	AAT->CAT	N->H	NP->BP
G	Unknown	23,932	22.47	3	Ac34	TAT->CAT	Y->H	NP->BP
T	Unknown	23,938	22.88	3	Ac34	GAG->AAG	E->K	AP->BP
C	Unknown	24,013	22.45	3	Ac34	CGC->GGC	R->G	BP->No polarity
G	Unknown	24,025	22.20	3	Ac34	AAT->CAT	N->H	NP->BP
G	Unknown	24,112	24.62	3	Ac34	TAT->CAT	Y->H	NP->BP
G	Unknown	24,205	24.73	3	Ac34	GGG->CGG	G->R	No polarity->BP
G	Unknown	24,226	24.57	3	Ac34	GGG->CGG	G->R	No polarity->BP
G	Unknown	24,263	24.26	3	Ac34	GAT->GCT	D->A	AP->No polarity
C	Unknown	24,267	24.15	3	Ac34	AAT->AAG	N->K	NP->BP
T	Unknown	24,277	24.28	3	Ac34	CAG->AAG	Q->K	NP->BP
C	Unknown	41,973	34.83	4	Ac55	TTG->TCG	L->S	No polarity->NP
A	Unknown	73,784	25.98	3	Ac91	CCA->TCA	P->S	No polarity->NP
T	Unknown	74,061	15.53	3	Ac91	TTA->TAA	L-> *	No polarity->None
A	Unknown	89,718	14.28	3	Ac106/107	CCA->ACA	P->T	No polarity->NP
G	Unknown	93,822	20.30	3	Ac114	AAT->CAT	N->H	NP->BP
C	Unknown	133,289	37.26	4	Ac7/orf603	CTG->CGG	L->R	No Polarity->BP
T	Unknown	133,648	37.68	4	Ac7/orf603	CCA->ACA	P->T	No polarity->NP
G	Unknown	133,708	26.94	3	Ac7/orf603	AAC->CAC	N->H	NP->BP
C	Unknown	133,738	27.70	4	Ac7/orf603	AAG->GAG	K->E	BP->AP

^a^ Nucleotide; ^b^ Gene function based on [54] and [62], PA: Packaging and Assembly; ^c^ Position of the SNP on WP10 consensus genome. Variations less than 260 bases distant are found linked in the pairs of reads; ^d^ Percentage of the SNP in WP10 genome population; ^e^ Gene ORF based name/gene alternative names. Based on [27], genes essential for the baculovirus are underlined; ^f^ Amino acid (AA) class changes based on [53], Classification III by charge and polarity, AP: Acidic and polarity, BP: Basic and polarity, NP: Neutral and polarity; * Stop codon

Interestingly, we found SNPs changing amino-acid polarity in a number of essential core genes and core functions. We detected mutations in several genes involved in transcription, including three subunits of the RNA polymerase (*lef-8*, *lef-9* and *lef-4*). Similarly, *lef-9* was linked with different transmission phenotypes in Spodoptera exigua NPV [54]. Altogether this highlights the potential key role of transcription in the regulation of the infection cycle and intra-cellular adaptation. We also found high frequency SNPs in four genes (*pif-1, pif-2*, *pif-3* and *vp91*) encoding essential components of the *per os* infectivity complex, which binds to insect midgut cells and is therefore involved in primary infection [55]. We might hypothesize that the presence of different forms of the PIF proteins in the WP10 population might allow the binding of this cell entry complex to an increased number of cell types, which could be advantageous for a generalist virus such as AcMNPV. However, there might be alternative explanations to this polymorphism. In Spodoptera frugiperda NPV, *pif-1* over-expression is detrimental to viral population fitness and leads to increased frequency of genotypes lacking *pif-1* expressing capabilities to regulate the amount of PIF-1 protein available in the cell [56]. Such compensatory mechanism might also be at play in AcMNPV populations.

As cells are commonly multiply infected during a baculovirus infection [17,24,57], beneficial high frequency SNPs are likely co-expressed with the consensus in infected cells. This may lead to different types of protein interaction, such as functional competition or complementation. For instance, *gp64* (*Ac128*), which is the envelope fusion protein, is oligomerized during transport to the plasma membrane [58]. When oligomerization is disrupted, the GP64 protein fails to accumulate at the cell surface thus impeding the spread of secondary infections [59]. Genetic variation could interfere in the formation and stability of GP64 oligomers. Slightly modified proteins in a high amount in the population might modulate these essential viral functions, and modify the course of infection.

Transmission of baculoviruses is generally achieved after the disintegration of insect cuticle mediated by viral chitinase and cathepsin [60]. We found seven highly frequent SNPs in the *Chitinase* (*Ac126*) (Table 3). This could have consequences on the interactions with the cathepsin, but also on the timing of chitinase release from the endoplasmic reticulum [61] and efficacy of host liquefaction. Of note our analyses revealed that *Ac7, Ac34* and *Ac91,* three genes of unknown function, have several high frequency SNPs (Table 3); one of *Ac91* SNPs, representing 16% of the population, encode a stop codon, drastically reducing the protein sequence from 223 to 19 amino acids. Functional studies on this gene are needed to assess how this SNP could impact viral fitness.

Like a magnet for finding a needle in a haystack, the k-mean clustering allowed us to point out the most significant protein variants in our viral population, *i.e*., 60 non-synonymous SNPs of the clusters 3 and 4 among 3243 SNPs. This is in large contrast with the extent of variation present in the population. When considering the SNPs from cluster 1, which represent the vast majority (78%) of all SNPs, 52% are non-synonymous but are in extremely low frequency in the population (0.12%), and thus appear functionally negligible.

To further assess under which process our baculovirus population evolved, we ran a homemade script estimating Tajima’s D for every positions of the genome. The estimated mean Tajima’s D was −0.55, which represents a population evolving close to the mutation drift equilibrium [47]. Deleterious mutations are usually eliminated and thus less likely to be sampled in a given population than neutral mutations. In the case of baculoviruses, cells are commonly multiply infected [19] and the virions transmitted in groups within the occlusion bodies (Figure 1). This reduces the purifying selection on single genomes within the population because deleterious mutations can be maintained through complementation [23,24]. This may explain why the mean Tajima’s D is slightly under 0. Most AcMNPV-WP10 genome positions are very close to this mean (sd = 0.14) and are thus not adaptive. This result echoes the neutral theory of evolution that posits the vast majority of variations are not adaptive and occur by chance [2]. In contrast, when we focused on the SNPs in cluster 3 and 4 that changed the amino acid polarity (Table 3), we found a mean Tajima’s D of 1.89 (sd = 0.66). These positions, in minority in the population, deviate from mutation drift equilibrium. As their Tajima’s D statistics are higher than 0 and significantly different from the genome mean (Welch two sample *t*-test, *p* < 2.2 × 10^−6^), they therefore evolve under balancing selection. This supports our interpretation that these highly frequent SNPs are involved in the adaptive process of our viral population.

### 3.3. Characterization of Large Deletions

**Figure 5:**
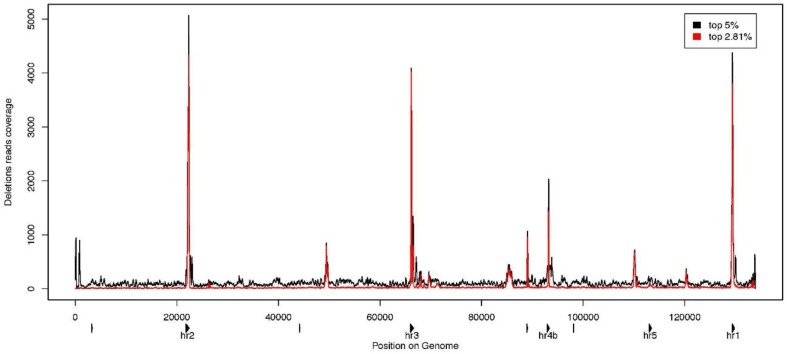
Deletion reads coverage along the AcMNPV-WP10 genome. The 5% (in black) and 2.81% (in red) reads presenting the highest pair distance were mapped on the genome. The coverage by these reads is shown all along the WP10 consensus genome, revealing deletion hotspots. The triangles represent the positions of the *hrs* on the genome. *Hrs* with their name under the triangle have been studied in more depth. The 2.81% reads are more distant than 669 nt that is the length of *hr2*, the largest *hr*. By comparing these two sets, we show that not only deletions of palindrome repeats in the *hrs* are present in the population, but also larger deletions, possibly occurring between two *hrs*.

As genomes harboring large deletions have been shown to bring synergistic fitness effects to some baculovirus populations [24], we endeavored to determine if our WP10 genome population contained such large deletions. We thus developed a new approach to find large indels in our genome population. The principle consists in re-mapping all paired-end reads as single-end reads on the WP10 consensus genome, and in comparing the mapping distance between each pair of paired-end reads with the expected distance as defined by the size of the sequencing insert (Figure 2). If the reads are closer than expected, they come from a genome where there are more bases between the two reads of a pair than measured on the consensus genome, so there is an insertion in this particular genome. In contrast, if they are more distant, they come from a genome where they are closer, so there is a deletion between them (Figure 2). Unfortunately, we could not use this strategy to find large insertions because the 260 bp insert produced using Illumina strategy is shorter than the 2 × 151 bp reads. Few large insertions have however been detected in this dataset by another strategy based on the analysis of chimeric reads [34]. In contrast, the strategy was quite efficient in identifying large deletions, outlined by pairs of paired-end reads more distantly mapped than expected. The 5% reads farthest to the mean distance were mapped to determine the location and repartition of the biggest deletions in the genome (Figure 5). We found big deletions across the entire genome. However some regions appeared more prone to deletions as we obtained coverage spikes in homologous regions, *i.e*., the *hrs* 1, 2, 3, 4b and 5.

There are eight *hrs* in AcMNPV. These regions contain repeated 70 bp units with an imperfect 30 bp palindrome in their center [27]. As *hrs* are highly recombinogenic and highly variable in size, we investigated the possibility that our observations could be artifactual by excluding all pairs of reads less distant than the largest *hr* size (669 bp for *hr2*). By mapping the 2.81% more distant pairs of reads, we excluded deletions shorter than 669 bp and thus removed those deleting the *hrs* only. As we still have spikes in the same regions as when mapping the 5% most distant pairs of reads (Figure 5), we thus confirmed that large deletions are not just deleting *hrs*, but do extract large sequences around these *hrs*, spanning coding sequences. The deletion between *hr5* and *hr1* was confirmed by PCR amplification and re-sequencing showing our strategy could outline genuine deletions, but it has not yet been possible to confirm the existence of the other large deletions. The *hr5* to *hr1* region spans 16,858 bp in the consensus genome and contains 19 genes, including early and late essential genes (six early expressed genes: *p26*, *Ac145*, *Ac146*, *ie-1*, *ie-2* and *pe38*; twelve late expressed genes: *p10*, *p74*, *me53*, *ie-0*, *49K*, *odv-e18*, *odv-ec27*, *odv-e56*, *Ac149*, *Ac150*, *Ac152* and *Ac154*; and *Ac140*) [49]. These deleted genomes are therefore not able to replicate on their own. The occluded structure of baculovirus infectious particles allows the maintenance of genomes with deleterious mutations in the virus population [23,24]. As we found large deletions everywhere on the consensus genome, no portion of the genome seems protected from deletions. These large deletions do not appear to dramatically impact the overall fitness of the viral population even though they are present in a fairly large proportion of genomes (25.47%).

Interestingly, most large deletions seemed anchored on *hrs* (number 1, 2, 3, 4b and 5), which have been proposed to serve as replication origins [63], and their number correlated to replication efficiency [64]. Baculovirus DNA is thought to replicate by rolling circle followed by extensive recombination [65]. The replication thus induces a highly recombinogenic state [27] potentially allowing the deletion of large parts of the genome, such as those we observed. However, the question of whether recombination between *hrs* is involved in the formation of these deletions remains open. For these deletion mutants to replicate though, they would need to have conserved a replication origin. This is a prerequisite for their maintenance and selection within the viral population. Alternatively, the deleted genome we found could result from badly resolved replication and random encapsidation within OBs. In AcMNPV, the adaptiveness of these deletion mutants has yet to be investigated. Deletion between *hrs* means genes are deleted as loci related sets, regardless of their function or whether they are essential. Each deletion mutant therefore needs to be complemented to complete its infection cycle. This would appear as a burden to the population, unless these mutants, by replicating more rapidly, could somehow enhance the replication of the whole virus population. For example, they might accelerate the production of proteins from the early release of RNA matrix.

## 4. Conclusions

The real extent of genetic variation in populations remains barely known although it is expected to greatly impact adaptation to the environment. To test this, we studied the genetic diversity of a generalist dsDNA virus population. Based on our ultra-deep sequencing data, we showed that variability is widely present in our AcMNPV genome population, both in the form of SNPs and of large deletions. Defective interfering (D.I.) particles, produced during viral replication are well known in RNA and DNA viruses [66,67]. Since D.I. particles require “helper” (complete) genomes to complete their cycles, they are commonly considered to have a negative effect on virus populations [66]. In contrast, a certain frequency of defective genomes has been shown to exert a synergistic effect on baculovirus populations [24]. Of note, the large deletions are found here in the same proportion as the optimal proportion of defective genomes in Lopez-Ferber’s studies [24]. The same type of synergistic interaction between D.I. and complete dengue viral particles were recently shown to increase viral transmission through the attenuation of disease symptoms [68]. Mutated and defective genomes thus appear important for the adaptation of baculoviruses to their hosts, but functional studies are required to determine their specific role.

The evolutionary arms race [69] occurs at several levels in the case of baculoviruses. Selection applies to virus populations, to OBs, to virions and even to individual genomes at the scale of individual infected cells, of systemic infection of one host and of successful transmission to a second host. OBs have been shown to foster the maintenance of genetic variants [57]. Here we displayed the extent of the genetic variation baculovirus can rely upon in the evolutionary arms race. First, the presence of numerous SNPs allows the dynamic selection of the fittest genomes within infected cells. Second, large deletion mutants, which themselves could harbor SNPs, could participate in the process of group selection [56]. The encapsidation within OBs of numerous and genetically diverse baculovirus genomes should bring a certain level of pre-adaptation to baculovirus populations. In bacteria, high mutation frequency due to mutator genes has been shown to accelerate adaptation [70]. Likewise, RNA viruses transmitted as genetically diverse populations present a higher fitness than populations transmitted through bottlenecks [71]. Ultra-deep sequencing gives unprecedented insights on the genetic variability present within a large dsDNA virus population. To test the adaptiveness of highly variable viral populations though, one would need to study their evolution in different ecological conditions and to model the sequence space occupied in different niches [72].

## Supplementary Files

Supplementary File 1

## Data Deposition

The raw data have been deposited at Genbank under the accession SRS533250 and the AcMNPV-WP10 sequence under the accession number KM609482.
