# Supplementary material for: Ultra Deep Sequencing of a Baculovirus Population Reveals Widespread Genomic Variations
Source: Viruses. 2015 Jul 7;7(7):3625–46. doi: 10.3390/v7072788 (PMC4517117; doi:10.3390/v7072788)
Supplement: Supplementary File 1 [file viruses-07-02788-s001.pdf]

Supplementary Material

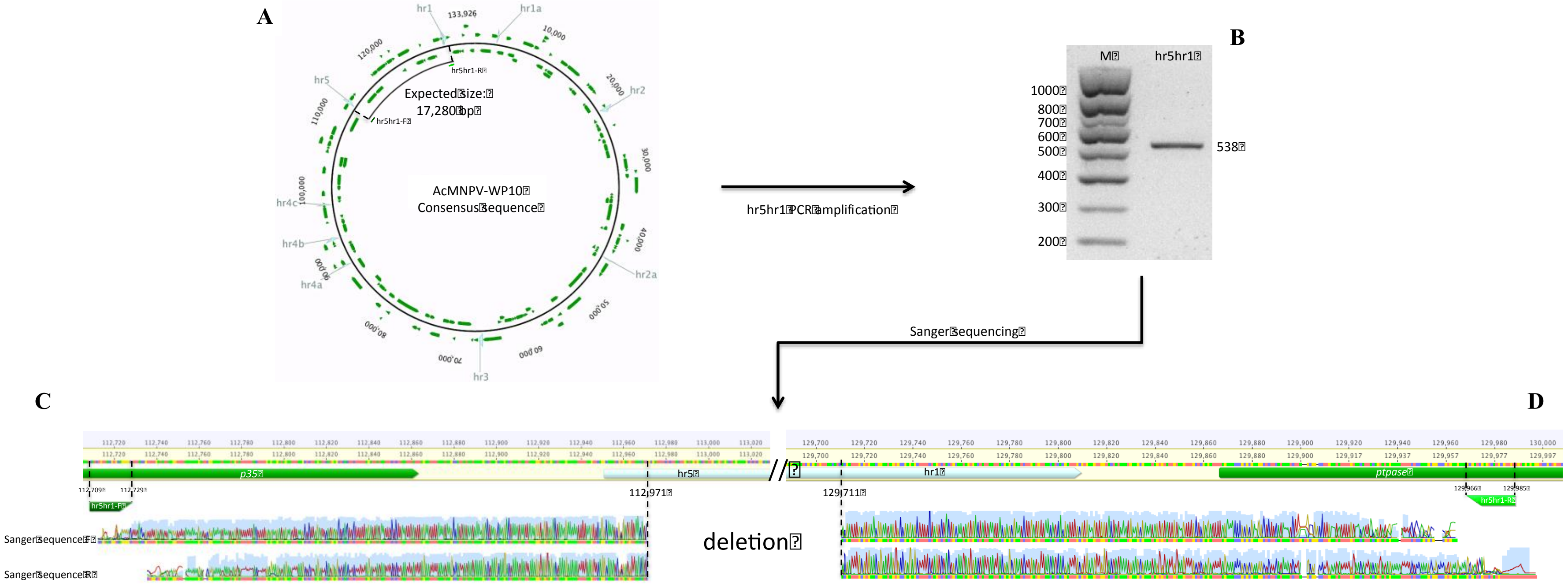

Figure S1. Map of the limits of the deletion between hr5 and hr1 found in AcMNPV wild population by PCR. This figure represents the circular map of the complete AcMNPV-WP10 consensus sequence (**A**), the hr5hr1 PCR amplification profile (**B**), the map of the genome at the location of p35 and hr5 (**C**) and of hr1 and ptpase (**D**). On the circular map (**A**), green arrows represent genes and light-blue arrows represent hrs. The amplified sequence is deported and the two green lines called hr5hr1-F and hr5hr1-R represent the position of the primers. After PCR amplification with the hr5hr1 primers the amplicon migrated on a 1.2% GelRed (Interchim) agarose electrophoresis gel with SmartLadderSF (Eurogentec) (**B**). On graphs (**C**) and (**D**) is represented the consensus sequence with the annotation, under it are the forward and reverse primers designed in the gene before hr5 and after hr1, which were used to perform the PCR confirming the presence of the deletion (Primer hr5hr1-F: CTACAGAATCGAGCTGGGGC; Primer hr5hr1-R: TCTTCGCTAGTCACGTACGC). The electrophoregrams represent the forward and reverse sanger sequence of PCR product spanning the deletion. The large gap observed when mapping the Sanger sequences on the consensus genome sequence confirms the presence of the deletion between hr1 and hr5. The deletion is located between the positions 112,971 and 129,711 of the consensus.
